# Supplementary material for: Removal of promoter CpG methylation by epigenome editing reverses HBG silencing
Source: Nat Commun. 2025 Jul 27;16:6919. doi: 10.1038/s41467-025-62177-z (PMC12297318; doi:10.1038/s41467-025-62177-z)

Supplementary Data

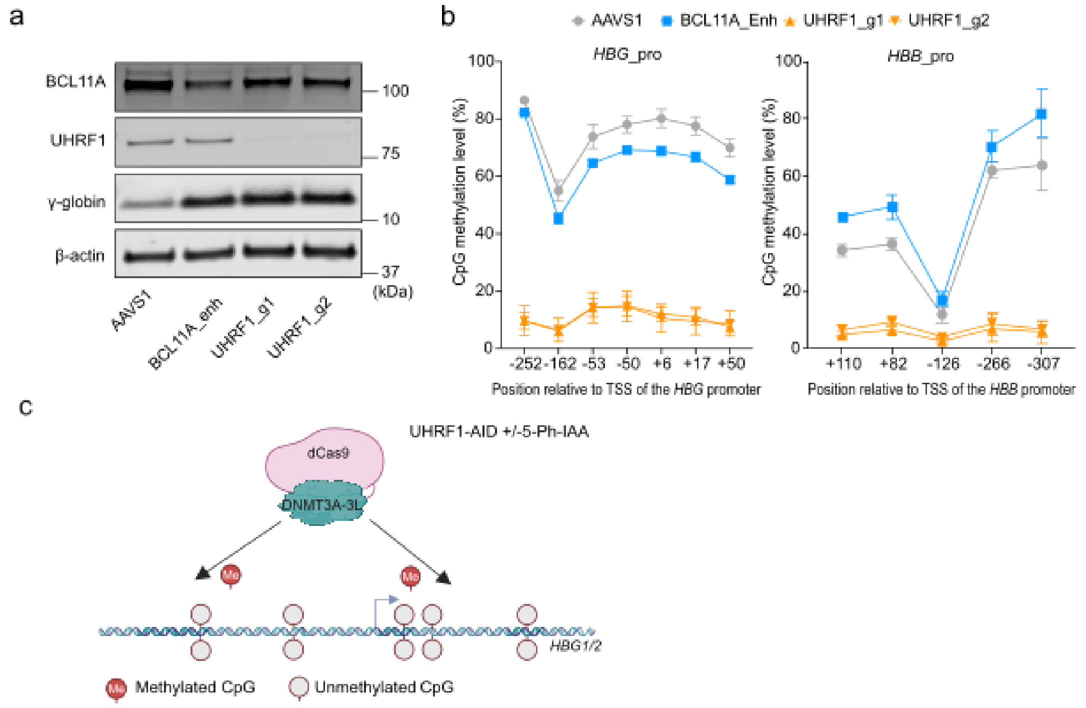

Supplementary Fig. 1. *UHRF1* KO induces *HBG* expression. a. Western blot shows BCL11A, UHRF1, γ-globin and β-actin in in erythroblasts derived from CD34^{+} HSPCselectroporated with BCL11A enhancer and UHRF1 sgRNA 1, 2**. b.** Amplicon bisulfite sequencing results showing the methylation level at *HBG* (left panel) and *HBB* (right panel) gene promoter CpG sites. Graph shows the mean percentage ± s.d. of three biological replicate experiments. **c**. Experimental strategy of enforced methylation of the *HBG* promoter in UHRF1-degraded and -restored HUDEP2 cells. Created in BioRender. Feng, R. (2025) https://BioRender.com/9qayfxx.

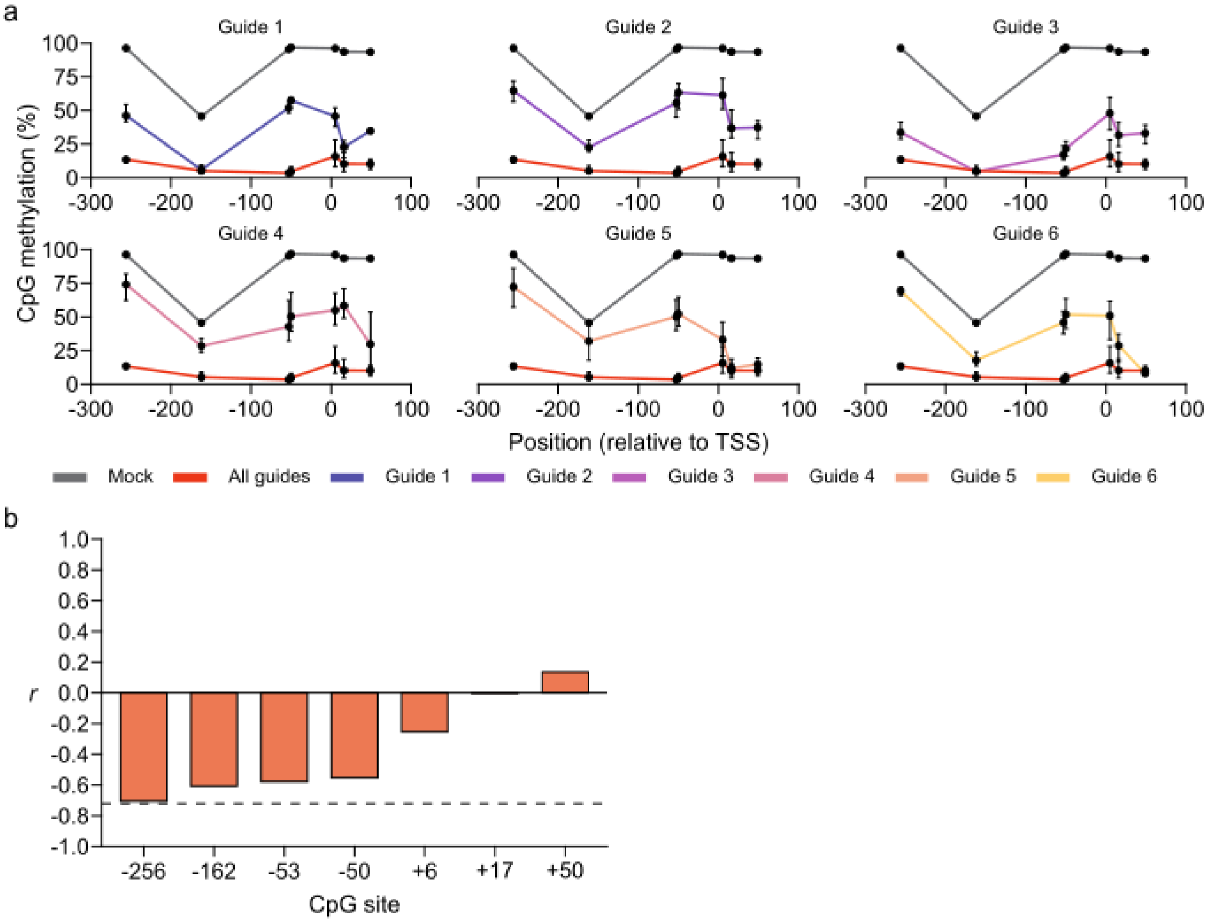

Supplementary Fig. 2. TETv4 treatment with individual guides causes partial demethylation of the *HBG* promoter. a. Amplicon bisulfite sequencing of *HBG* promoter CpG methylation in HUDEP2 cells treated with TETv4 and six targeting guides applied individually. Upper (grey) and lower (orange) lines represent CpG methylation percentage in mock and TETv4 *HBG* (all guide) treatments, respectively. Points indicate the mean methylation of three technical replicates. Error bars represent range. **b.** Pearson's correlation (*r*) between the CpG methylation percentage at individual sites within the *HBG* proximal promoter from a. and %*HBG/(HBG+HBB)* from Fig. 4b across HUDEP2 cells treated with TETv4 and individual guides targeting the *HBG* promoter. Horizontal dashed line represents the Pearson's correlation coefficient calculated from the mean methylation of CpG sites upstream of the TSS from Fig. 4c.

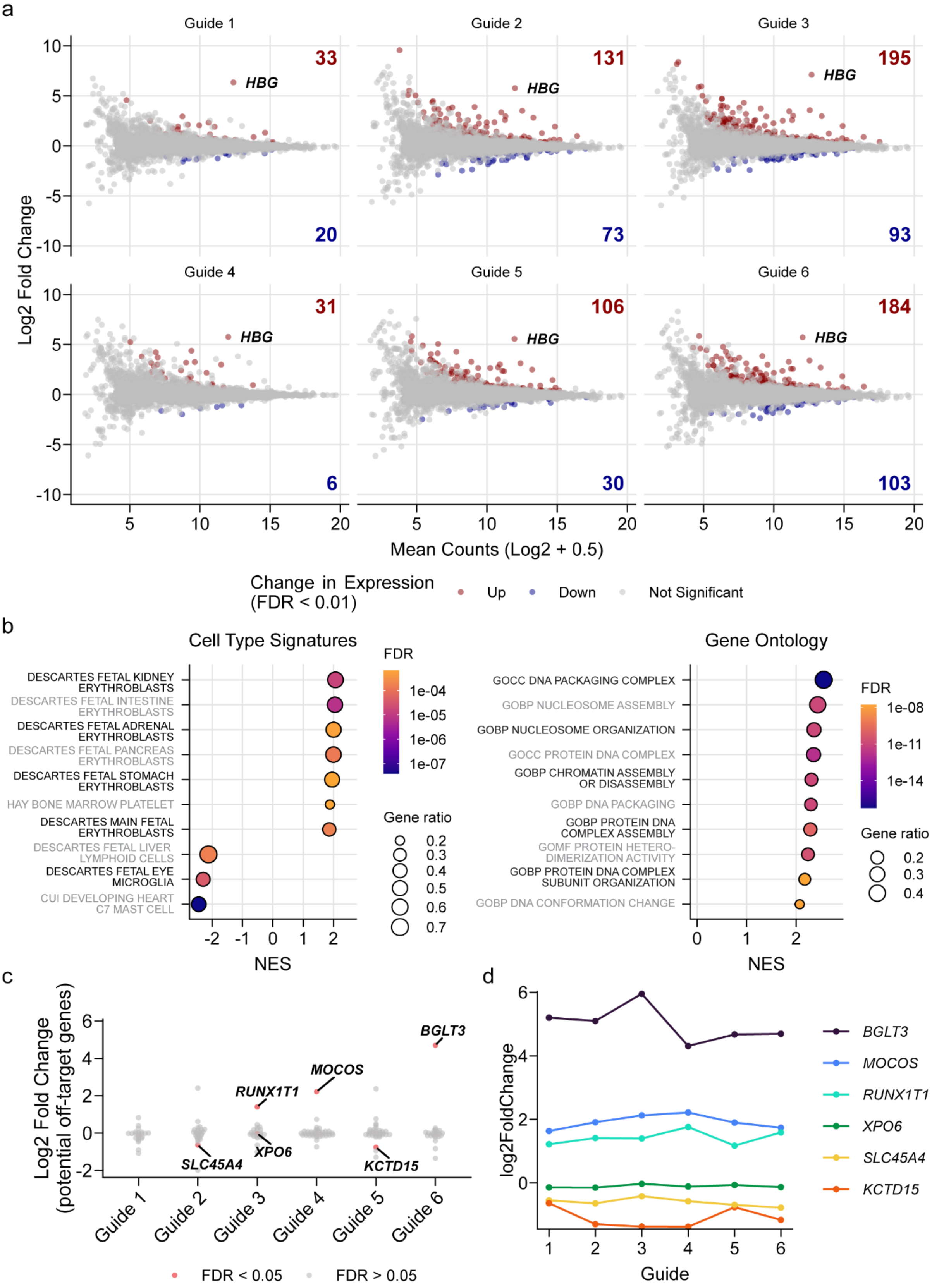
a
Guide 1
Guide 2
Guide 3
Guide 4
Guide 5
Guide 6
HBG
33
20
131
73
195
93
31
6
106
30
184
103
Log2 Fold Change
Mean Counts (Log2 + 0.5)
Change in Expression (FDR < 0.01)
Up
Down
Not Significant
b
Cell Type Signatures
DESCARTES FETAL KIDNEY ERYTHROBLASTS
DESCARTES FETAL INTESTINE ERYTHROBLASTS
DESCARTES FETAL ADRENAL ERYTHROBLASTS
DESCARTES FETAL PANCREAS ERYTHROBLASTS
DESCARTES FETAL STOMACH ERYTHROBLASTS
HAY BONE MARROW PLATELET
DESCARTES MAIN FETAL ERYTHROBLASTS
DESCARTES FETAL LIVER LYMPHOID CELLS
DESCARTES FETAL EYE MICROGLIA
CUI DEVELOPING HEART C7 MAST CELL
NES
FDR
1e-04
1e-05
1e-06
1e-07
Gene ratio
0.2
0.3
0.4
0.5
0.6
0.7
Gene Ontology
GOCC DNA PACKAGING COMPLEX
GOBP NUCLEOSOME ASSEMBLY
GOBP NUCLEOSOME ORGANIZATION
GOCC PROTEIN DNA COMPLEX
GOBP CHROMATIN ASSEMBLY OR DISASSEMBLY
GOBP DNA PACKAGING
GOBP PROTEIN DNA COMPLEX ASSEMBLY
GOMF PROTEIN HETERO-DIMERIZATION ACTIVITY
GOBP PROTEIN DNA COMPLEX SUBUNIT ORGANIZATION
GOBP DNA CONFORMATION CHANGE
FDR
1e-08
1e-11
1e-14
Gene ratio
0.2
0.3
0.4
c
Log2 Fold Change (potential off-target genes)
BGLT3
MOCOS
RUNX1T1
XPO6
SLC45A4
KCTD15
FDR < 0.05
FDR > 0.05
d
log2FoldChange
Guide
BGLT3
MOCOS
RUNX1T1
XPO6
SLC45A4
KCTD15

Supplementary Fig. 3. TETv4 treatments in HUDEP2 cells promote erythroid differentiation signatures. a. MA plots of differentially expressed genes in HUDEP2 lines treated with TETv4 and individual *HBG* guides *vs*. mock (3 technical replicates per group). Colored points/labels represent genes identified as upregulated (red) or downregulated (blue) with a false discovery rate (FDR) cutoff of 1% (Benjamini-Hochberg method). *HBG* represents transcripts mapping to both *HBG1* and *HBG2*. **b.** Gene set enrichment analysis of cell type signatures (left) and gene ontology (right) in TETv4 *HBG*-treated *vs*. mock HUDEP2 cells. FDR = false discovery rate (Benjamini-Hochberg method). NES = normalised enrichment score. Gene ratio represents the proportion of the gene set that is present in the dataset. **c.** Log2 fold changes of genes adjacent to predicted off-target sites of individual *HBG* guides in HUDEP2 cells treated with the respective guide compared to mock. Red points represent significantly differentially expressed genes at a false discovery rate cutoff of 5% (Benjamini-Hochberg method). **d.** Log2 fold changes of differentially expressed genes identified in c. across all six guides.

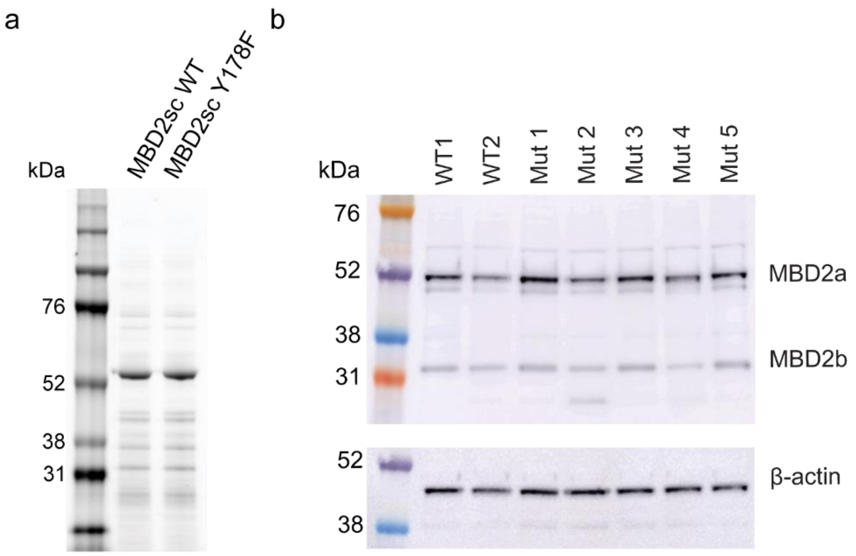

Supplementary Fig. 4. MBD2 Y178F is expressed similarly to WT MBD2. a. Coomassie stain of expressed MBD2sc WT and Y178F protein prepared for use in EMSA. **b.** Western blot showing MBD2a (top) and MBD2b (middle) protein expression in five HUDEP2 MBD2 Y178F mutant clones compared to WT, with β-actin loading control (bottom). kDa = kilodalton. Western blot was replicated once with similar results.

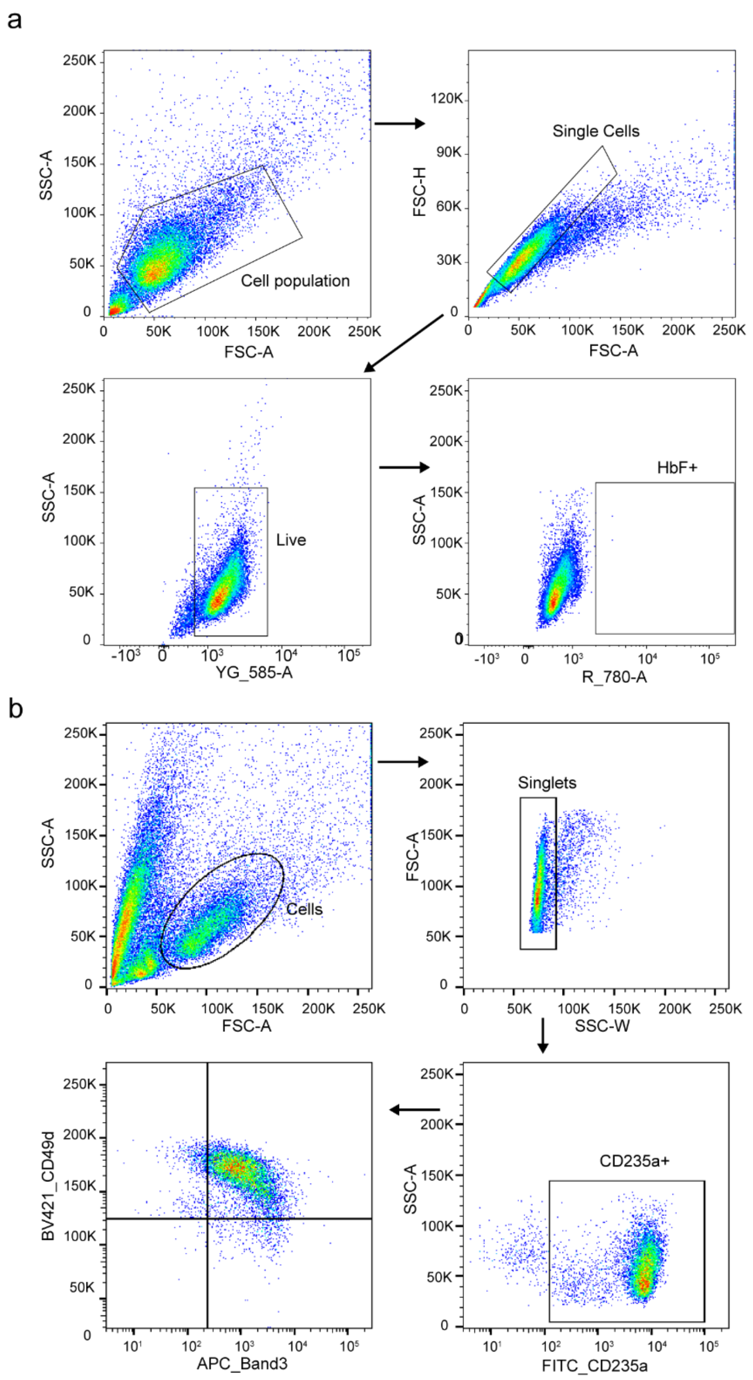

Supplementary Fig. 5. **Representative gating strategy for detection of HbF-APC+ stained HUDEP2 cells.** Arrows indicate the order of gates applied. Labels indicate target population. SSC = side scatter, FSC = forward scatter. Final panel corresponds to data panel in manuscript. **a.** Gating strategy for Figure 3e. **b.** Gating strategy for Figure 5d.

Supplementary Table 1. Guide sequences used in epigenome editing experiments

Name	Position (centre of guide to gene TSS)	Sequence
HBG guide 1	-184	ATTTGCATTGAGATAGTGTG
HBG guide 2	-145	CCATGGGTGGAGTTTAGCCA
HBG guide 3	-110	CTTGACCAATAGCCTTGACA
HBG guide 4	+12	ACTCGCTTCTGGAACGTCTG
HBG guide 5	+59	CGCCATGGGTCATTTCACAG
HBG guide 6	+90	GCTACTATCACAAGCCTGTG
EPX guide 1	-226	CCACTGATGCACTTCTCCCA
EPX guide 2	-90	GTTTCAGAACTCCACCCAAG
EPX guide 3	+39	GGGGGTCCTCAAAGTGAGAG
EPX guide 4	+121	GCAGATGCATCTCTGCAGCG

Supplementary Table 2. Primer sequences used to prepare amplicons for bisulfite sequencing and in vitro transcription

Name	Orientation	Sequence
HBG1 proximal promoter and UTR	Forward	AAAGGGAAGAATAAATTAGAGAAAAACTGG
HBG2 proximal promoter and UTR	Forward	AAAGGGAAGAATAAATTAGAGAAAAATTGG
HBG proximal promoter and UTR	Reverse	AACCTTATCCTCCTCTATAAAATAACCCAT
HBG distal promoter	Forward	ATTTTTGAAAAGTTTAGGGATTGTGAAATG
HBG1 distal promoter	Reverse	CACACACACTTATCCAATAATAAATACACATCAT
HBG2 distal promoter	Reverse	CACATACACTTATCCAATAATAAATACACATCAT
**Epigenome editing tools IVT template amplification (HUDEP2)**	Forward	TAATACGACTCACTATAAGCCTACAGCTCCTGGGCAAC
**Epigenome editing tools IVT template amplification (HUDEP2)**	Reverse	GCCACCACCTTCTGATAGGC
Epigenome editing tools IVT template amplification ($CD34^+$)	Forward	GCAAAGAATTCTAATACGACTCACTATAAGCTCATCATTTTGGCAAAGAATTCTG
Epigenome editing tools IVT template amplification ($CD34^+$)	Reverse	TTTTTTTTTTTTTTTTTTTTTTTTTTTTTTTTTTTTTTT<br>TTTTTTTTTTTTTTTTTTTTTTTTTTTTTTTTTTTTTTT<br>TTTTTTTTTTTTTTTTTTTTTTTTTTTTTTTTTTTTTTT<br>TTTGATGCTCAAGGGGCTTCATGATGTC

Supplementary Table 3. Primer sequences used in detection of cDNA by qPCR

Name	Orientation	Sequence
18S cDNA	Forward	CACGGCCGGTACAGTGAAAC
18S cDNA	Reverse	AGAGGAGCGAGCGACCAA
HBG cDNA	Forward	CCTGTCCTCTGCCTCTGCC
HBG cDNA	Reverse	GGATTGCCAAAACGGTCAC
HBB cDNA	Forward	TGTCCACTCCTGATGCTGTTATG
HBB cDNA	Reverse	GGCACCGAGCACTTTCTTG

Supplementary Table 4. Oligonucleotide sequences used in genome editing

Name	Description	Sequence
MBD2 +643A>T donor	Single strand donor oligo homologous to MBD2 with A>T substitution (red) at position +643 downstream of the *MBD2* TSS.	GGCCCTCCCCCCCGGATGGAAGAA GGAGGAAGTGATCCGAAAATCTGG GCTAAGTGCTGGCAAGAGCGATGT CTTCTACTTCAGGTACCTCCCTGGG GGCGGGGAGGGGGTGGCGGGGTC AGGCCGGGGTCAGGGGTCAAGAG CGGGCCT
MBD2 +647 guide	Guide sequence for cutting MBD at position +647	AGAGCGATGTCTACTACTTC
UHRF1 sgRNA1	Guide sequence for editing UHRF1	CTACAACCCCGACAACCCCA
UHRF1 sgRNA1	Guide sequence for editing UHRF1	ACACCCGACTCGCTGACCTG
BCL11A enhancer sgRNA	Guide sequence editing BCL11A enhancer	CTAACAGTTGCTTTTATCAC

Supplementary Table 5. Antibodies used in CUT&RUN experiments

Name	Catalog #	Vendor
BCL11A	ab191401	Abcam
GATA1	ab11852	Abcam
NFYA	ab6558	Abcam
H3K4me3	ab8580	Abcam
H3K9ac	9649	Cell Signaling Technology
H3K27ac	ab4729	Abcam

Supplementary Table 6. Oligonucleotide sequences used in EMSA

Name	Orientation	Sequence
**BCAT1 promoter CpG sites**	Sense	CCGGCCCTCTCGCGGCGGAGACTCGCGACCTAGCGGATTGCATCAGCAGGAAGAC
**BCAT1 promoter CpG sites**	Antisense	GTCTTCCTGCTGATGCAATCCGCTAGGTCGCGAGTCTCCGCCGCGAGAGGGCCGG
**BCAT1 promoter CpG sites methylated**	Sense	C/Me-dC/GGCCCTCT/Me-dC/G/Me-dC/GG/Me-dC/GGAGACT/Me-dC/G/Me-dC/GACCTAG/Me-dC/GGATTGCATCAGCAGGAAGAC
**BCAT1 promoter CpG sites methylated**	Antisense	GTCTTCCTGCTGATGCAATC/Me-dC/GCTAGGT/Me-dC/G/Me-dC/GAGTCTC/Me-dC/GC/Me-dC/G/Me-dC/GAGAGGGC/Me-dC/GG
**HBG -53/-50 CpG sites**	Sense	TGAGGCCAGGGGCCGGCGGCTGGCTAGGGAT
**HBG -53/-50 CpG sites**	Antisense	ATCCCTAGCCAGCCGCCGGCCCCTGGCCTCA
**HBG -53/-50 CpG sites methylated**	Sense	TGAGGCCAGGGGC/Me-dC/GG/Me-dC/GGCTGGCTAGGGAT
**HBG -53/-50 CpG sites methylated**	Antisense	ATCCCTAGCCAGC/Me-dC/GC/Me-dC/GGCCCCTGGCCTCA

Supplementary Raw Data

Supplementary Fig. 1a

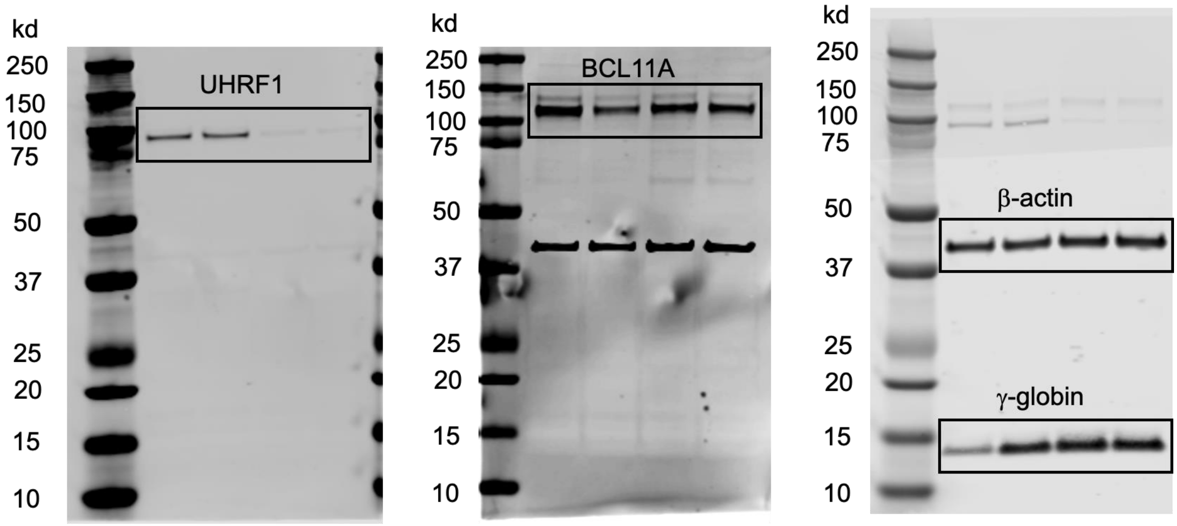

Supplementary Fig. 1b

HBG promoter

	AAVS1			BCL11A_Enh			UHRF1_g1			UHRF1_g2		
-252	86.26804	87.63188	85.52702	82.05492	82.12529	82.34103	7.607333	12.90467	8.279963	15.68777	7.188021	6.201263
-162	56.08998	57.87609	50.9327	45.91116	46.73016	43.10562	5.864729	8.066494	4.465117	11.10594	4.941287	3.493487
-53	75.82754	76.62847	68.97429	64.96281	65.4938	63.41848	15.21212	16.99518	10.65331	19.99875	13.14562	9.638318
-50	79.3083	80.15509	74.75268	69.33635	70.15438	67.95015	15.9268	17.66031	10.88382	20.37357	13.63418	9.963318
+6	81.53553	82.44001	76.50626	69.64753	69.71539	67.07553	11.54749	14.78286	9.874177	16.16653	8.098054	7.289742
+17	78.63534	80.04688	73.96338	67.55403	67.83506	64.84247	10.71847	13.68142	8.404604	15.02164	7.23665	6.298333
+50	70.5383	72.71538	66.48337	59.29978	59.94854	57.22364	6.745427	9.771521	5.994371	13.78588	6.616097	5.773252

HBB promoter

	AAVS1			BCL11A_Enh			UHRF1_g1			UHRF1_g2		
+110	32.96242	33.28455	36.49543	44.62134	46.95792	46.18028	5.784705	4.206578	3.127418	7.729724	5.398641	5.634882
+82	38.24048	37.28899	33.73603	45.88771	48.00275	54.45414	7.224476	5.708725	3.968919	10.117	8.402191	5.901354
-126	11.58938	15.10421	8.987947	15.1804	19.85277	15.93973	2.243846	2.91094	2.108589	4.461681	3.85131	3.671535
-266	58.93187	62.46225	64.75952	74.67144	72.45283	64.41569	9.278769	9.862645	1.603898	10.75282	4.3842	10.7627
-307	56.1719	61.50338	73.8759	75.33464	78.84625	91.10907	7.13542	8.967339	1.459683	8.44901	3.328616	8.539189

Supplementary Fig. 2a

Replicate	1	2	3	1	2	3	1	2	3	1	2	3	1	2	3	1	2	3	1	2	3	1	2	3
Position	Mock			TETv4 HBG			Guide 1			Guide 2			Guide 3			Guide 4			Guide 5			Guide 6		
50	93.331	92.704	94.249	13.614	10.52	6.1398	34.404	35.752	34.123	42.193	28.805	40.865	25.241	34.737	39.083	22.103	53.604	13.404	11.098	19.517	13.626	9.8081	6.3587	8.0014
17	93.27	93.563	93.896	18.654	7.2226	4.5709	14.892	25.898	27.484	50.408	29.538	30.321	23.296	30.316	40.93	49.122	70.962	54.801	7.1138	15.057	13.354	37.104	16.618	32.054
6	96.102	95.482	96.855	28.051	11.289	8.1189	38.025	46.961	51.974	73.715	50.905	59.063	35.592	48.635	59.613	53.223	67.525	43.936	20.78	31.922	46.098	57.878	33.12	61.662
-50	96.881	96.83	96.696	3.6647	7.9755	2.2618	57.097	56.086	59.379	69.089	50.942	70.043	16.024	21.788	26.931	39.176	68.057	43.64	43.032	49.059	64.739	41.296	50.3	63.683
-53	95.363	95.532	95.447	2.3008	5.7101	2.1885	51.438	48.171	56.214	61.236	44.934	60.419	13.209	14.729	23.686	33.776	62.255	32.197	39.767	48.383	62.528	46.251	37.319	54.052
-162	45.722	44.848	46.064	8.903	3.5383	3.0172	3.2345	4.9757	9.2072	27.754	20.258	19.353	3.5671	3.5164	6.3155	23.751	33.802	27.523	17.857	47.296	30.568	15.749	23.86	13.639
-256	96.271	96.126	96.501	14.626	14.406	11.108	54.495	41.495	42.555	71.782	56.517	65.775	31.037	28.703	41.171	78.133	81.979	62.163	73.562	57.172	85.977	71.98	69.668	65.983

Supplementary Fig. 4a

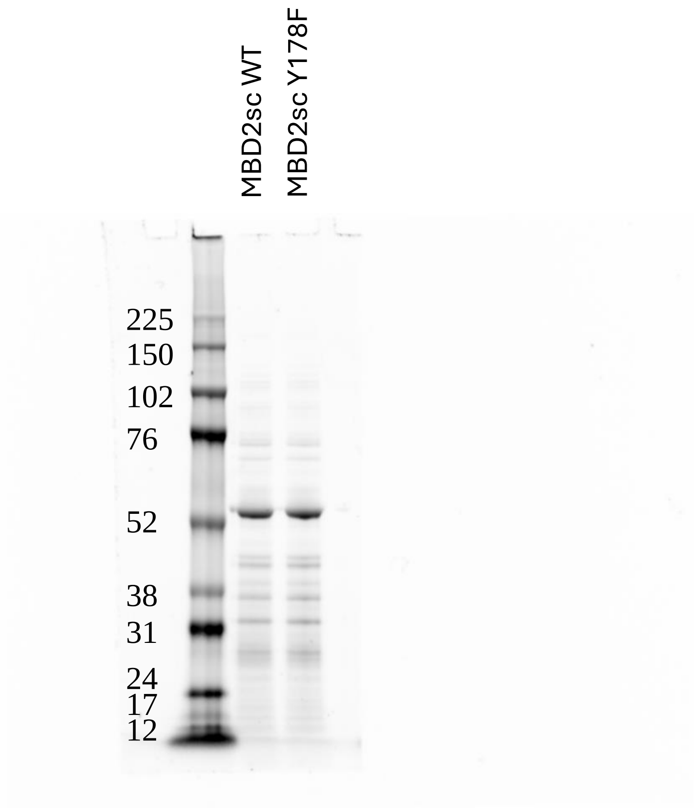

Supplementary Fig. 4b

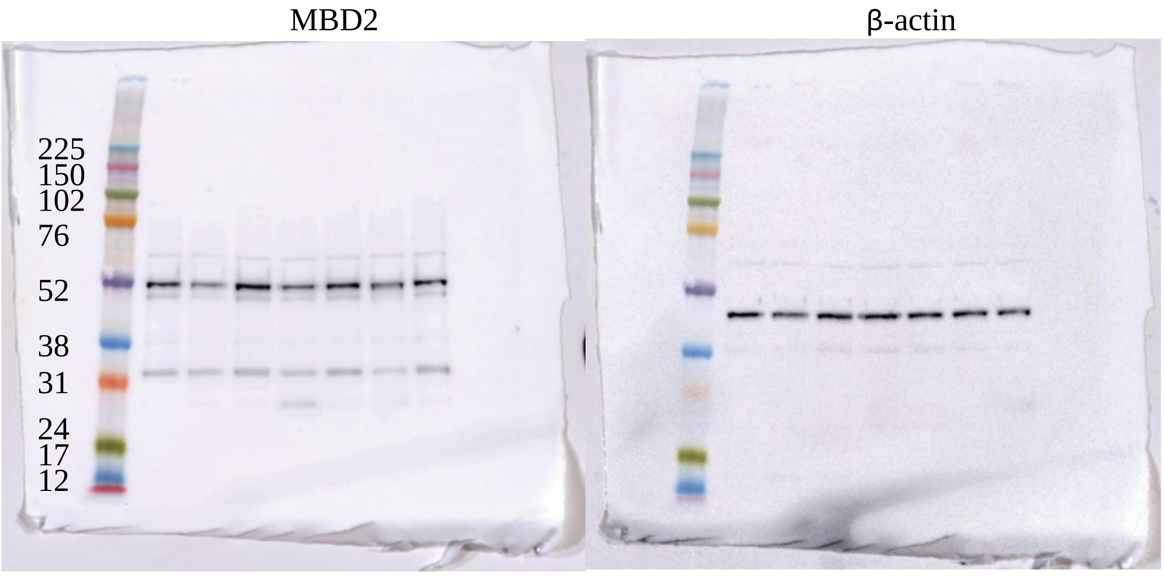

Supplement: Supplementary file 1 — Supplementary Information [file 41467_2025_62177_MOESM1_ESM.pdf]
