# Supplementary material for: Removal of promoter CpG methylation by epigenome editing reverses HBG silencing
Source: Nat Commun. 2025 Jul 27;16:6919. doi: 10.1038/s41467-025-62177-z (PMC12297318; doi:10.1038/s41467-025-62177-z)
Supplement: Supplementary file 3 — Reporting Summary [file 41467_2025_62177_MOESM3_ESM.pdf]

nature portfolio

Corresponding author(s): Merlin Crossley
Mitchell J. Weiss

Last updated by author(s): 2025/04/11

Reporting Summary

Nature Portfolio wishes to improve the reproducibility of the work that we publish. This form provides structure for consistency and transparency in reporting. For further information on Nature Portfolio policies, see our Editorial Policies and the Editorial Policy Checklist.

Statistics

For all statistical analyses, confirm that the following items are present in the figure legend, table legend, main text, or Methods section.

n/a	Confirmed	
☐	☒	The exact sample size (*n*) for each experimental group/condition, given as a discrete number and unit of measurement
☐	☒	A statement on whether measurements were taken from distinct samples or whether the same sample was measured repeatedly
☐	☒	The statistical test(s) used AND whether they are one- or two-sided<br>*Only common tests should be described solely by name; describe more complex techniques in the Methods section.*
☒	☐	A description of all covariates tested
☐	☒	A description of any assumptions or corrections, such as tests of normality and adjustment for multiple comparisons
☐	☒	A full description of the statistical parameters including central tendency (e.g. means) or other basic estimates (e.g. regression coefficient) AND variation (e.g. standard deviation) or associated estimates of uncertainty (e.g. confidence intervals)
☒	☐	For null hypothesis testing, the test statistic (e.g. *F*, *t*, *r*) with confidence intervals, effect sizes, degrees of freedom and *P* value noted<br>*Give P values as exact values whenever suitable.*
☒	☐	For Bayesian analysis, information on the choice of priors and Markov chain Monte Carlo settings
☒	☐	For hierarchical and complex designs, identification of the appropriate level for tests and full reporting of outcomes
☐	☒	Estimates of effect sizes (e.g. Cohen's *d*, Pearson's *r*), indicating how they were calculated

Our web collection on statistics for biologists contains articles on many of the points above.

Software and code

Policy information about availability of computer code

Data collection	qPCR data was collected using QuantStudio Real Time PCR software v1.3 or QuantStudio 6/7 Pro Software v1.8.0<br>Western blot images were captured using ImageQuant LAS 500 software v1.1.0<br>Flow cytometry data was collected using BD FACS Diva software v9<br>RNAseq data was collected on Illumina Miseq, Nextseq, and Novaseq instruments by the Ramaciotti Centre for Genomics with default software.<br>CUT&RUN data was collected on Illumina Nextseq and Novaseq instruments by Hartwell center in St. Jude with default setting.
Data analysis	RNAseq data was analysed using the nf-core RNAseq workflow v3.14 and the Sleuth package v0.30.1 in R v4.4.1<br>Gene set enrichment analysis was performed using the msigdbr package v7.5.1 and clusterProfiler package v4.12.6 in R v4.4.1<br>Matching of potential off target sites to nearby genes was performed using the GenomicRanges package v1.58.0 in R v 4.4.1<br>CUT&RUN raw FASTQ data were mapped to hg19 by using BWA-MEM (version 0.7.16a). Reads that could not be uniquely mapped to the human genome were removed by SAMtools (version 0.17). Peaks were called by using MACS2 (version 2.1.1). BigWiggle files were generated using DeepTools(version 3.2.0).

For manuscripts utilizing custom algorithms or software that are central to the research but not yet described in published literature, software must be made available to editors and reviewers. We strongly encourage code deposition in a community repository (e.g. GitHub). See the Nature Portfolio guidelines for submitting code & software for further information.

Data

Policy information about availability of data

All manuscripts must include a data availability statement. This statement should provide the following information, where applicable:

- Accession codes, unique identifiers, or web links for publicly available datasets
- A description of any restrictions on data availability
- For clinical datasets or third party data, please ensure that the statement adheres to our policy

All raw and processed high throughput sequencing data have been deposited in the NCBI GEO under accession number GSE284289,GSE284290,GSE284291.

Research involving human participants, their data, or biological material

Policy information about studies with human participants or human data. See also policy information about sex, gender (identity/presentation), and sexual orientation and race, ethnicity and racism.

Reporting on sex and gender	*Use the terms sex (biological attribute) and gender (shaped by social and cultural circumstances) carefully in order to avoid confusing both terms. Indicate if findings apply to only one sex or gender; describe whether sex and gender were considered in study design; whether sex and/or gender was determined based on self-reporting or assigned and methods used.*<br>*Provide in the source data disaggregated sex and gender data, where this information has been collected, and if consent has been obtained for sharing of individual-level data; provide overall numbers in this Reporting Summary. Please state if this information has not been collected.*<br>*Report sex- and gender-based analyses where performed, justify reasons for lack of sex- and gender-based analysis.*
Reporting on race, ethnicity, or other socially relevant groupings	*Please specify the socially constructed or socially relevant categorization variable(s) used in your manuscript and explain why they were used. Please note that such variables should not be used as proxies for other socially constructed/relevant variables (for example, race or ethnicity should not be used as a proxy for socioeconomic status).*<br>*Provide clear definitions of the relevant terms used, how they were provided (by the participants/respondents, the researchers, or third parties), and the method(s) used to classify people into the different categories (e.g. self-report, census or administrative data, social media data, etc.)*<br>*Please provide details about how you controlled for confounding variables in your analyses.*
Population characteristics	*Describe the covariate-relevant population characteristics of the human research participants (e.g. age, genotypic information, past and current diagnosis and treatment categories). If you filled out the behavioural & social sciences study design questions and have nothing to add here, write "See above."*
Recruitment	*Describe how participants were recruited. Outline any potential self-selection bias or other biases that may be present and how these are likely to impact results.*
Ethics oversight	*Identify the organization(s) that approved the study protocol.*

Note that full information on the approval of the study protocol must also be provided in the manuscript.

Field-specific reporting

Please select the one below that is the best fit for your research. If you are not sure, read the appropriate sections before making your selection.

☒ Life sciences ☐ Behavioural & social sciences ☐ Ecological, evolutionary & environmental sciences

For a reference copy of the document with all sections, see nature.com/documents/nr-reporting-summary-flat.pdf

Life sciences study design

All studies must disclose on these points even when the disclosure is negative.

Sample size	Sample sizes of 2-3 replicates were used for experiments in HUDEP2 and CD34+ cells. This sample size was determined based on expectations of the field, sufficiency for detecting the effect of treatment, and the practicality of generating and maintaining the total number of samples per experiment.
Data exclusions	MBD2 Y178F mutant HUDEP2 cells were excluded based on abnormal MBD2 expression in pilot assays. An equal number of wild type control clones were selected for comparison in RNAseq based on proximity to the mean expression of gamma globin for this group in pilot qPCR analysis.
Replication	Epigenome editing in HUDEP2 cells were replicated 2-3 times by repeat treatment of independent samples derived from the same parent population and processed in parallel. Epigenome editing in CD34+ cells was replicated 3 times using 3 independent patient derived samples. Genome editing in HUDEP2 cells were replicated 2-5 times by sampling of independent biological clones from the edited population. All attempts at replication were successful.

Randomization	Randomised allocation to treatment groups was not relevant to this study. Treated samples were compared to mock treated populations or clones derived from the same parent population.
Blinding	Investigators were not blinded to sample identity. Data was produced using objective quantitative methods to remove the influence of subjective bias.

Reporting for specific materials, systems and methods

We require information from authors about some types of materials, experimental systems and methods used in many studies. Here, indicate whether each material, system or method listed is relevant to your study. If you are not sure if a list item applies to your research, read the appropriate section before selecting a response.

Materials & experimental systems

n/a	Involved in the study
☐	☒ Antibodies
☐	☒ Eukaryotic cell lines
☒	☐ Palaeontology and archaeology
☒	☐ Animals and other organisms
☒	☐ Clinical data
☒	☐ Dual use research of concern
☒	☐ Plants

Methods

n/a	Involved in the study
☐	☒ ChIP-seq
☐	☒ Flow cytometry
☒	☐ MRI-based neuroimaging

Antibodies

Antibodies used	Anti-MBD2, Abcam, ab188474, clone EPR18361<br>Goat Anti-Rabbit IgG H&L (HRP), abcam, ab205718, polyclonal<br>Anti-β-actin, Sigma-Aldrich, A1978, clone AC-15<br>Sheep anti-Mouse IgG, HRP-linked whole Ab, Cytiva, NA931, polyclonal<br>Anti-HbF APC, Invitrogen, #MHFH05, clone HBF-1<br>BCL11A, Abcam, ab191401<br>GATA1, Abcam, ab11852<br>NFYA, Abcam, ab6558<br>H3K4me3, Abcam, ab8580<br>H3K9ac, Cell Signaling Technology,9649<br>H3K27ac, Abcam, ab4729<br>CD49d, Biolegend, 304322<br>CD235a,eBioscience, 11-9886-42<br>Band3 Gift from Xiuli An
Validation	All antibodies were acquired from commercial suppliers with validation performed by the supplier.<br>Fluorescent antibodies were validated by the suppliers through flow cytometry against isotype controls (PMID: 23422750).

Eukaryotic cell lines

Policy information about cell lines and Sex and Gender in Research

Cell line source(s)	HUDEP2 cells were obtained from Ryo Kuritam Cell engineering division, RIKEN BioResource Centre, Japan. The sex of this cell line is male (XY).
Authentication	CD34+ cells were enriched via immunomagnetic bead selection using an Auto MACS instrument (Miltenyi Biotec). After enrichment, the CD34+ cell fraction was >95% of the total, with <0.2% CD3+ cells and <0.1% CD19+ cells.<br>HUDEP2 cell lines were authenticated by qPCR to confirm the low-HBG phenotype prior to their use in experiments.
Mycoplasma contamination	Cell lines were periodically tested for mycoplasma and tested negative.
Commonly misidentified lines<br>(See ICLAC register)	No commonly misidentified cell lines were used in this study.

Plants

Seed stocks

Report on the source of all seed stocks or other plant material used. If applicable, state the seed stock centre and catalogue number. If plant specimens were collected from the field, describe the collection location, date and sampling procedures.

Novel plant genotypes

Describe the methods by which all novel plant genotypes were produced. This includes those generated by transgenic approaches, gene editing, chemical/radiation-based mutagenesis and hybridization. For transgenic lines, describe the transformation method, the number of independent lines analyzed and the generation upon which experiments were performed. For gene-edited lines, describe the editor used, the endogenous sequence targeted for editing, the targeting guide RNA sequence (if applicable) and how the editor was applied.

Authentication

Describe any authentication procedures for each seed stock used or novel genotype generated. Describe any experiments used to assess the effect of a mutation and, where applicable, how potential secondary effects (e.g. second site T-DNA insertions, mosiacism, off-target gene editing) were examined.

ChIP-seq

Data deposition

- [x] Confirm that both raw and final processed data have been deposited in a public database such as GEO.
- [ ] Confirm that you have deposited or provided access to graph files (e.g. BED files) for the called peaks.

Data access links
May remain private before publication.

https://www.ncbi.nlm.nih.gov/geo/query/acc.cgi?acc=GSE284291

Files in database submission

TETv4_BCL11A_R1.fastq.gz
TETv4_BCL11A_R2.fastq.gz
TETv4_GATA1_R1.fastq.gz
TETv4_GATA1_R2.fastq.gz
TETv4_H3K4me3_R1.fastq.gz
TETv4_H3K4me3_R2.fastq.gz
TETv4_NFY_R1.fastq.gz
TETv4_NFY_R2.fastq.gz
dTETv4_BCL11A_R1.fastq.gz
dTETv4_BCL11A_R2.fastq.gz
dTETv4_GATA1_R1.fastq.gz
dTETv4_GATA1_R2.fastq.gz
dTETv4_H3K4me3_R1.fastq.gz
dTETv4_H3K4me3_R2.fastq.gz
dTETv4_NFY_R1.fastq.gz
dTETv4_NFY_R2.fastq.gz
CD34_NT_BCL11A_R1.fastq.gz
CD34_NT_BCL11A_R2.fastq.gz
CD34_NT_GATA1_R1.fastq.gz
CD34_NT_GATA1_R2.fastq.gz
CD34_NT_H3K27ac_R1.fastq.gz
CD34_NT_H3K27ac_R2.fastq.gz
CD34_NT_H3K4me3_R1.fastq.gz
CD34_NT_H3K4me3_R2.fastq.gz
CD34_NT_H3K9ac_R1.fastq.gz
CD34_NT_H3K9ac_R2.fastq.gz
CD34_NT_NFYA_R1.fastq.gz
CD34_NT_NFYA_R2.fastq.gz
CD34_UHRF1gRNA1_BCL11A_R1.fastq.gz
CD34_UHRF1gRNA1_BCL11A_R2.fastq.gz
CD34_UHRF1gRNA1_GATA1_R1.fastq.gz
CD34_UHRF1gRNA1_GATA1_R2.fastq.gz
CD34_UHRF1gRNA1_H3K27ac_R1.fastq.gz
CD34_UHRF1gRNA1_H3K27ac_R2.fastq.gz
CD34_UHRF1gRNA1_H3K4me3_R1.fastq.gz
CD34_UHRF1gRNA1_H3K4me3_R2.fastq.gz
CD34_UHRF1gRNA1_H3K9ac_R1.fastq.gz
CD34_UHRF1gRNA1_H3K9ac_R2.fastq.gz
CD34_UHRF1gRNA1_NFYA_R1.fastq.gz
CD34_UHRF1gRNA1_NFYA_R2.fastq.gz

Genome browser session
(e.g. UCSC)

Not applicable.

Methodology

Replicates

One experiment for each of the antibodies in each type of cell been performed.

Sequencing depth

CUT&RUN samples were sequenced with paired-end 2X50 or 2X75.
Samples Total Reads(M) Distinct Reads(M)
dTETv4 NFY 43597414 10016130
TETv4 NFY 59775104 16582258
dTETv4 BCL11A 36536124 15929721
TETv4 BCL11A 43510190 18346049
dTETv4 GATA1 51328775 24214889
TETv4 GATA1 54860146 30482056
dTETv4 H3K4me3 44887625 20940248
TETv4 H3K4me3 35249321 18849399
CD34 NT BCL11A 28091520 20889303
CD34 UHRF1gRNA1 BCL11A 34969833 28422223
CD34 NT NFYA 103429923 35948592
CD34 UHRF1gRNA1 NFYA 97932070 35601424
CD34 NT H3K27ac 76500524 62701030
CD34 UHRF1gRNA1 H3K27ac 69115145 58160749
CD34 NT H3K9ac 120013870 96561130
CD34 UHRF1gRNA1 H3K9ac 92390083 77723826
CD34 NT H3K4me3 98914310 44224050
CD34 UHRF1gRNA1 H3K4me3 101241610 51126719

Antibodies

BCL11A, Abcam, ab191401
GATA1, Abcam, ab11852
NFYA, Abcam, ab6558
H3K4me3, Abcam, ab8580
H3K9ac, Cell Signaling Technology,9649
H3K27ac, Abcam, ab4729

Peak calling parameters

Peaks were called by using MACS2 (version 2.1.1).

Data quality

Raw data was quality checked with FastQC.
Sample peak
dTETv4 NFY 25642
TETv4 NFY 23439
dTETv4 BCL11A 32525
TETv4 BCL11A 30632
dTETv4 GATA1 142854
TETv4 GATA1 125833
dTETv4 H3K4me3 33422
TETv4 H3K4me3 62515
CD34 NT BCL11A 21538
CD34 UHRF1gRNA1 BCL11A 9026
CD34 NT NFYA 49779
CD34 UHRF1gRNA1 NFYA 41573
CD34 NT H3K27ac 104548
CD34 UHRF1gRNA1 H3K27ac 83004
CD34 NT H3K9ac 43880
CD34 UHRF1gRNA1 H3K9ac 44683
CD34 NT H3K4me3 40472
CD34 UHRF1gRNA1 H3K4me3 56394

Software

CUT&RUN raw FASTQ data were mapped to hg19 by using BWA-MEM (version 0.7.16a). Reads that could not be uniquely mapped to the human genome were removed by SAMtools (version 0.17). BigWiggle files were generated using DeepTools(version 3.2.0).

Flow Cytometry

Plots

Confirm that:

- [x] The axis labels state the marker and fluorochrome used (e.g. CD4-FITC).
- [x] The axis scales are clearly visible. Include numbers along axes only for bottom left plot of group (a 'group' is an analysis of identical markers).
- [x] All plots are contour plots with outliers or pseudocolor plots.
- [x] A numerical value for number of cells or percentage (with statistics) is provided.

Methodology

Sample preparation	HUDEP2 cells were harvested from culture or thawed from cryopreserved samples stored at -80°C. Harvested cells were fixed using 0.05% glutaraldehyde and permeabilised with 0.1% Triton X-100 before staining with HbF-APC antibody (#MHFH05, Invitrogen) diluted 1:20 in PBS with 0.1% bovine serum albumin (BSA) for 17 minutes. Stained cells were resuspended in PBS with 0.1% BSA and analysed.
Instrument	LSRFortessa X-20 (BD Biosciences)
Software	BD FACSDiva (v9.0)
Cell population abundance	Pure HUDEP2 cell populations were used for F-cell staining experiments. >30 000 single cell events were obtained for each sample.
Gating strategy	Live HUDEP2 cells were initially gated from dead cells and debris based on forward scatter (FSC) and side scatter ratios (SSC). Single cells were gated from the parent population based on the linear relationship between FSC-A and FSC-H expected for singlets. Live cells were then gated from the parent population based off of detection of Kusabira orange marker expressed in live HUDEP2 cells on the yellow-green 585 nm wavelength. Finally, HbF+ cells were gated based off of the detection of APC on the red 780 nm wavelength, with the gate set to exclude the bulk of cells when not labelled with HbF-APC antibody.

- [x] Tick this box to confirm that a figure exemplifying the gating strategy is provided in the Supplementary Information.
